# A Comparison of Antifungal Drugs and Traditional Antiseptic Medication for Otomycosis Treatment: A Systematic Review and Meta-Analysis

**DOI:** 10.3389/fsurg.2021.739360

**Published:** 2021-12-22

**Authors:** Shunyu Wu, Yin Cheng, Shunzhang Lin, Huanhai Liu

**Affiliations:** Department of Otolaryngological, Shanghai Changzheng Hospital, Shanghai, China

**Keywords:** otomycosis, antifungal drug, traditional antiseptic medication, recovery rate, complication

## Abstract

**Objectives/Hypothesis:** To perform a systematic review and meta-analysis to compare the efficacy of and complications associated with antifungal drugs and traditional antiseptic medication for the treatment of otomycosis.

**Data Sources:** The PubMed, EMBASE, GeenMedical, Cochrane Library, CBM, CNKI, VIP and other databases were searched from January 1991 to January 2021.

**Methods:** The systematic literature review followed the Preferred Reporting Items for Systematic Reviews and Meta-Analyses (PRISMA) guidelines. Randomized controlled trials (RCTs) and non-randomized studies (case-control, cohort, and case series) were included to assess the topical use of antifungal drugs and traditional antiseptic medication in patients with otomycosis. The research subjects were patients who were clinically diagnosed with otomycosis and whose external auditory canal secretions were positive for fungi. Funnel plots were used to detect bias, and the Q test was used to assess heterogeneity. The random-effects model was used for meta-analysis. The *t*-test was used to assess significance.

**Results:** Of the 324 non-duplicate studies screened, 16 studies met the criteria for full-text review, and 7 were included in the meta-analysis. Four studies reported recovery conditions (*P* = 0.01). Six common complications after medication use were compared, and there were no significant differences. The authors further conducted subgroup analysis according to complications. The differences in the rates of ear distension (*P* = 0.007), earache (*P* = 0.03) and tinnitus (*P* = 0.003) were statistically significant.

**Conclusion:** The results of this meta-analysis and literature review showed that antifungal drugs and traditional antiseptic medication were effective in relieving symptoms in patients with otomycosis, and the two treatments were associated with different complications. Otolaryngologists have the option to use one medication or a combination of two drugs on the basis of the condition. Future research in this area should include RCTs with long-term follow-up to guide the development of otomycosis guidelines to overcome some of the weaknesses found in the literature.

**Systematic Review Registration:** https://www.crd.york.ac.uk/PROSPERO/#myprospero.

## Introduction

Otitis externa is a superficial fungal infection that can involve the middle ear, which is affected in more than 10% of all otitis externa cases (1). Bacterial infections of the ears are usually acute, while mycotic infections may be acute or subacute and cause inflammation and itching (2). Severe infections are generally caused by bacteria and may lead to the secretion of pus. As a chronic ear disease of the external auditory canal, the incidence of otomycosis is highest in the hot and humid seasons and lowest in the cold season. The most common pathogenic bacteria causing otomycosis are Candida, Aspergillus, Penicillium and so on, which lead to local histopathological changes (3). For example, in Aspergillus infections, bone invasion or tissue damage does not generally occur. The early stage of Candida infection is mainly characterized by exudation, and the late stage is characterized by granulomatous inflammation (4). Blastomyces and Actinomyces infections result in suppurative and granulomatous manifestations. Mucor invades blood vessels, causing thrombi, tissue infarction and leucocyte infiltration. Otomycosis can be asymptomatic, but fungi can multiply and accumulate to form masses that can block the external auditory canal, causing a sense of obstruction. Patients tend to experience a feeling of moisture in the external auditory canal because of fungal mass irritation, which causes a small amount of external auditory canal secretion. The blockage can worsen and invade the eardrum, leading to hearing impairment, tinnitus, and even dizziness (5). If the lesion increases in size, there may be local pain, and severe cases may cause facial paralysis (6).

For patients and physicians, otomycosis can be a very frustrating illness, although it is not a life-threatening ailment. Although otomycosis is not serious in most cases, immune compromise resulting from otomycosis is life-threatening to patients (7). The disease easily develops in patients in warm and humid environments, and it is difficult to obtain a good curative effect if patient treatment compliance is poor. Therefore, to ensure treatment adherence, topical drugs with simple application should be selected for treatment (8). Antifungal agents such as clotrimazole or terbinafine hydrochloride spray can be used, as well as traditional antiseptic medications such as povidone or boric acid alcohol (9). In general, systemic medication is unnecessary. In recent years, there have been great advances in antimicrobial therapy, though no widely accepted topical agent for the treatment of otomycosis has been developed.

Early detection of otomycosis and rapid intervention are important to reduce the negative effects of otomycosis on quality of life (10). Despite the availability of various interventions, their efficacy has rarely been compared, making it difficult to select an appropriate treatment. The authors conducted a systematic review incorporating pairwise and network meta-analyses (NMAs), with the objectives of assessing the relative effects of two medications in patients with otomycosis with regard to prognosis, complications and other key outcomes.

## Materials and Methods

### Search Strategy

The authors searched the PubMed, EMBASE, GeenMedical, Cochrane Library, CBM, CNKI, VIP and other databases using a combination of controlled vocabulary (e.g.,“Otomycosis,” “Fungus”) and keywords (e.g., “Therapeutic,” “Antifungal Drugs,” “Antiseptic Medication”). The databases were searched in May 2021, and articles related to drug treatment of fungal otomycosis that had been published or were in progress were extracted. In addition, the references of potentially relevant literature were screened.

### Eligibility Criteria

There were four criteria for inclusion. a. Research type: randomized controlled trial (RCT) or clinical Trial. b. Research subjects: patients who were clinically diagnosed with otomycosis and whose external auditory canal secretions were positive for fungi. c. Treatment method: topical drug treatment (the experimental group was treated with antifungal drugs, and the control group was treated with non-antifungal drugs). d. Research results: patient cure rate, symptom improvement, and complications after medication use (including ear itching, ear distension, ear pain, etc.).

The exclusion criteria were as follows. (a) Other diseases of the external auditory canal or middle ear with similar symptoms and imaging changes, such as external auditory canal osteoma, external auditory canal cholesteatoma, and middle ear spheroid tumor. (b) Surgical treatment or animal studies. (c) Duplicate studies. (d) Literature with incomplete data or no researchable indicators.

### Data Extraction

Two researchers extracted data from the included literature, and differences were resolved through discussion or the opinion of a third researcher. Extracted data included general information (first author, publication time, and country, sex, age) and clinical data (sample size of each group and corresponding rates of cure, symptom improvement, and complications).

### Risk of Bias Assessment

Since the included studies were mostly retrospective cohort studies, the Newcastle Ottawa Scale (NOS) was used to assess the risk of bias in the selection of study groups, comparability, and outcomes. A total of 7 items were evaluated, and each item received a score of “1” or “0.” The results are included in the basic research information and are displayed together.

### Data Analysis

RevMan5.4 software was used for meta-analysis, and *P* < 0.05 indicated that a difference was statistically significant. Odds ratios (ORs) and corresponding 95% confidence intervals (Cis) were used to analyse dichotomous variables, and the I^2^ statistic was used to assess heterogeneity. If *P* > 0.05 and I^2^ <50%, the included studies had no obvious heterogeneity, and the fixed-effect model was used. If *P* < 0.05 and I^2^ > 50%, the included studies had obvious heterogeneity, and the random-effects models was used; subgroup and sensitivity analyses were performed to explore the source of the heterogeneity. The analysis results are presented as forest plots, and publication bias is shown in funnel plots.

## Results

### General Study Information

**Figure 1:**
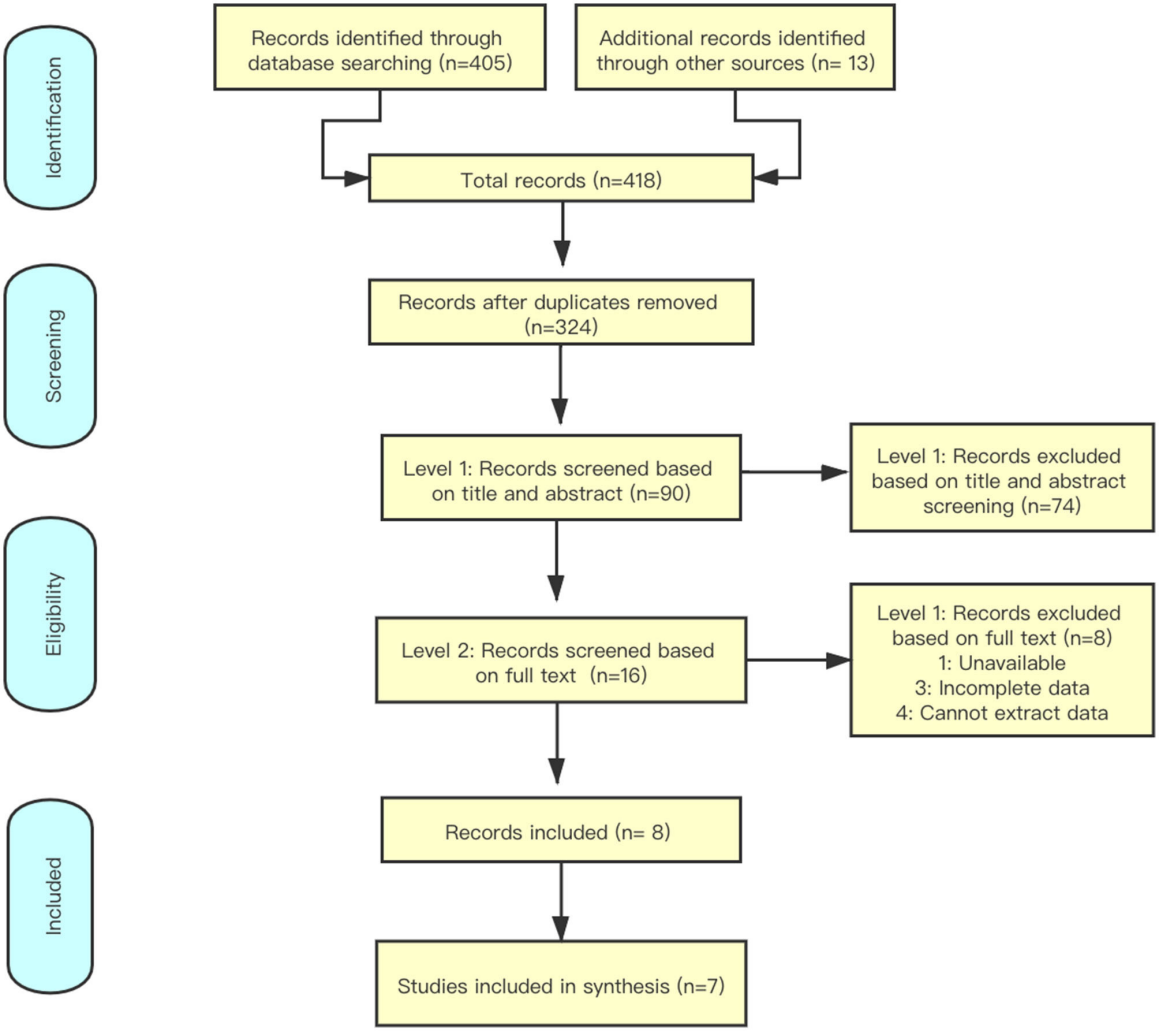
Preferred reporting items for systematic reviews and meta-analyses search strategy.

By searching the databases (Figure 1), 418 studies were identified, and 324 studies were selected for further screening. By reading the titles and abstracts of the literature, reviews, case reports or systematic reviews that did not meet the inclusion criteria were excluded. The remaining 16 articles were downloaded for full-text screening. Screening was strictly conducted according to the inclusion criteria. Studies with incomplete data or no research indicators were excluded, and 7 studies (Table 1) were finally included (11–17).

**Table 1:** Study characteristics and details of interventions.

**Author**	**Year**	**Region**	**Design**	**Mode of administration**		**Total N**	
				**Antifungal drug**	**Traditional antiseptic medication**	**Male**	**Female**
Boncalon et al.	2009	Philippines	RCT	Clotrimazole cream 1%	Kalachuchi Bark Extract Ointment	7	9
Mgbor and Gugnani	2001	Nigeria	RCT	Clotrimazole	Mercurochrome/ Locacorten-Vioform	38	34
Mofatteh et al.	2018	Iran	RCT	Clotrimazole	Betadine	86	118
Philip et al.	2013	India	RCT	1% Clotrimazole + Lignocaine	7.5% Povidone Iodine	17	17
Romsaithong et al.	2016	Thailand	RCT	1% Clotrimazole	3% Boric acid in 70% Alcohol	46	74
Swain et al.	2018	India	RCT	Clotrimazole	Povidone Iodine	19	25
Xu et al.	2020	China	RCT	Terbinafine Hydrochloride Spray + 3%BAA	3% Boric Acid Alcohol (BAA)	162	136

*RCT, randomized controlled trial*.

### Risk of Bias Assessment

**Figure 2:**
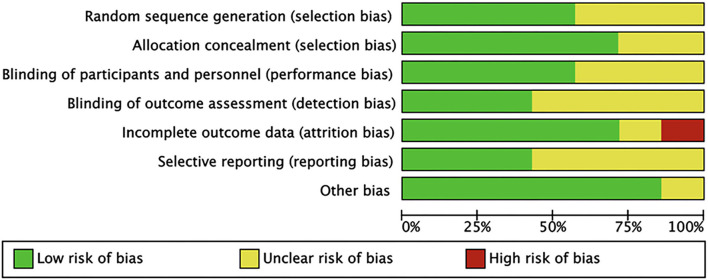
Risk of bias graph. Review authors' judgements about each risk of bias item presented as percentages across all included studies.

**Figure 3:**
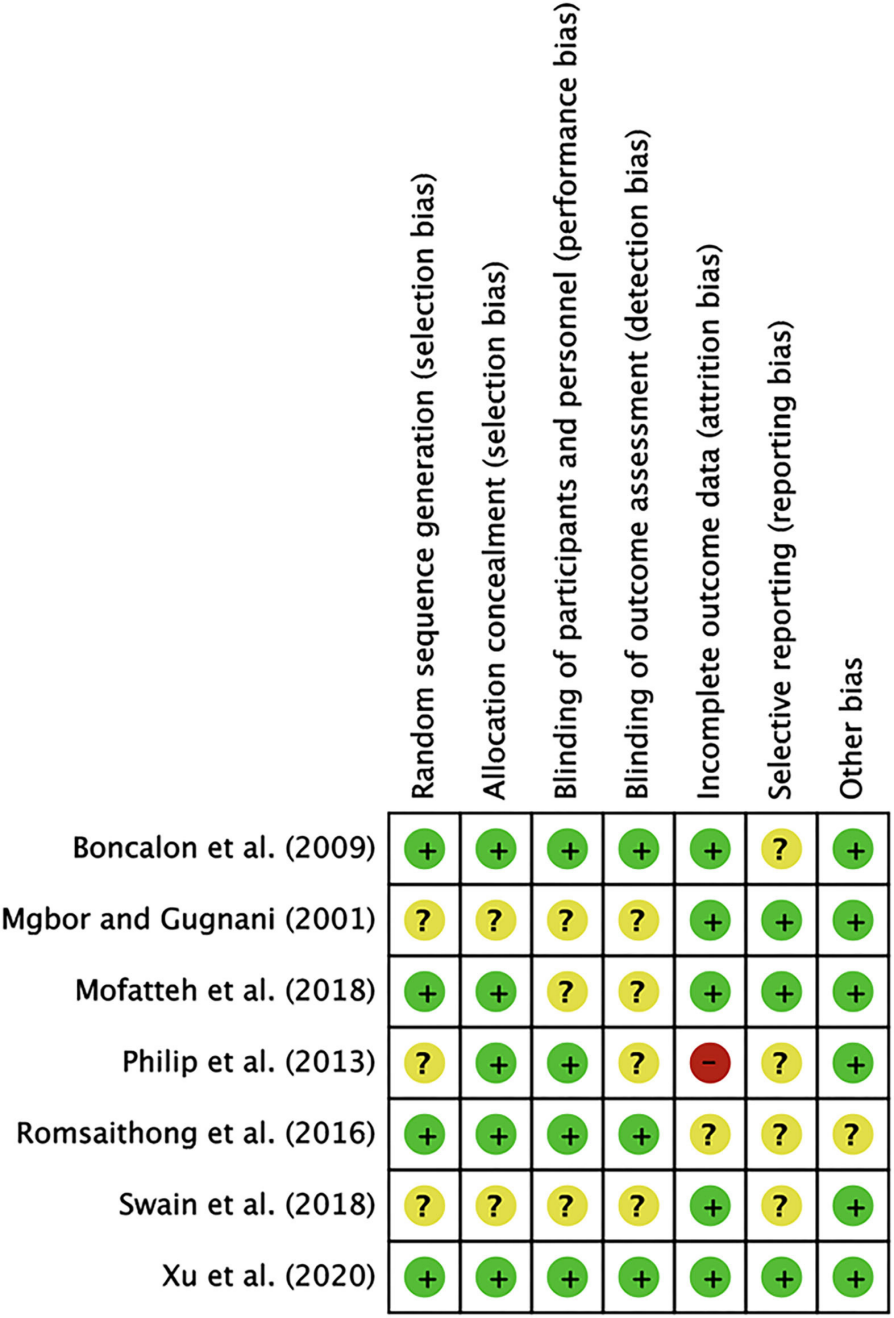
Findings of the risk of bias evaluation. Findings of the risk of bias evaluations using the Cochrane Scale are shown. Green, low risk of bias; red, high risk of bias; yellow, unclear risk of bias.

Based on 7 items, we estimated the quality of the literature. As summarized in Figure 1, only one study had a high risk of attrition bias. In this study, the follow-up patient data were incomplete, the number of people lost to follow-up was large, though the specific reasons were not explained. The last article was of higher quality because the information was more complete. Based on the above, all 7 articles were included in the meta-analysis (Figures 2, 3).

### Patient Population

**Figure 4:**
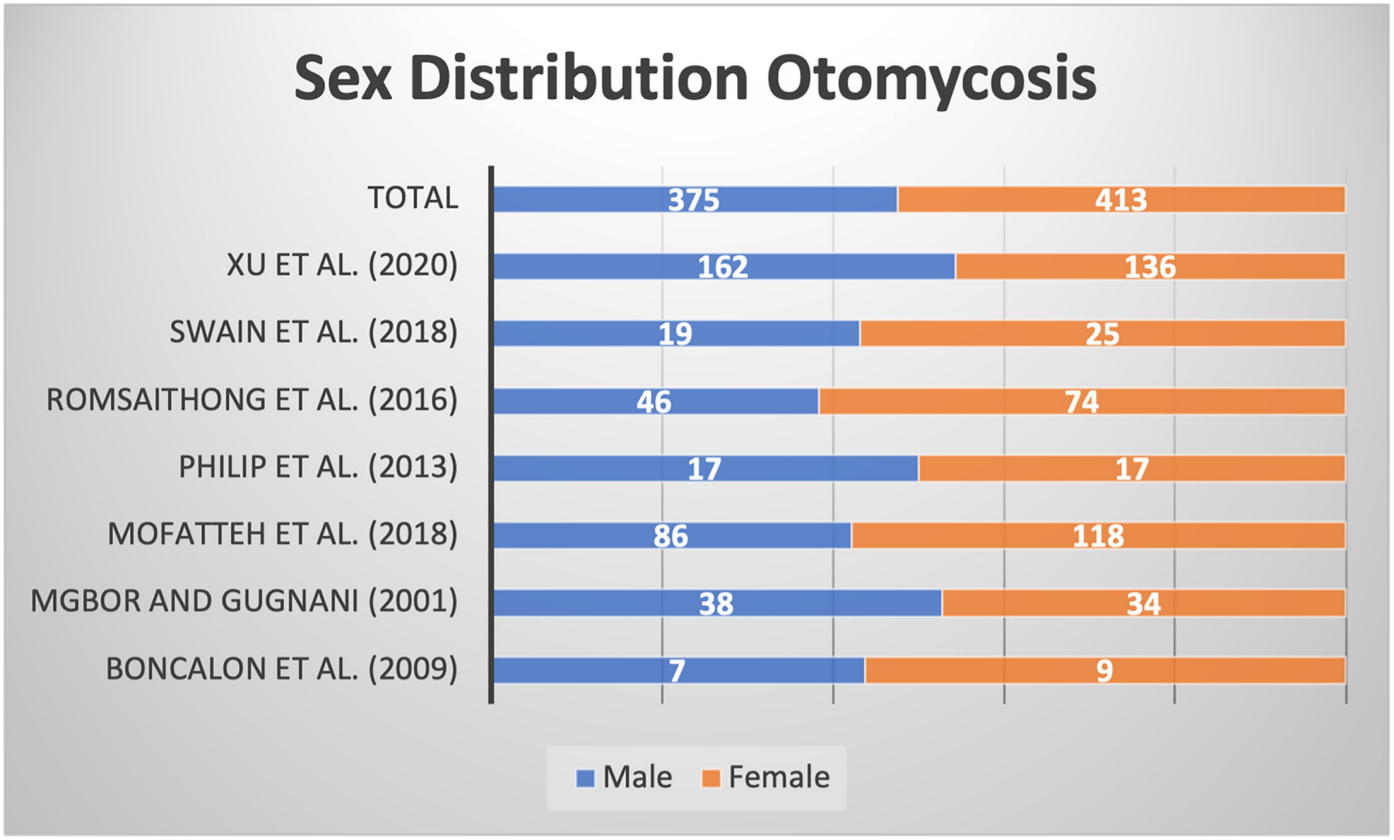
Sex ratios in the included studies.

**Figure 5:**
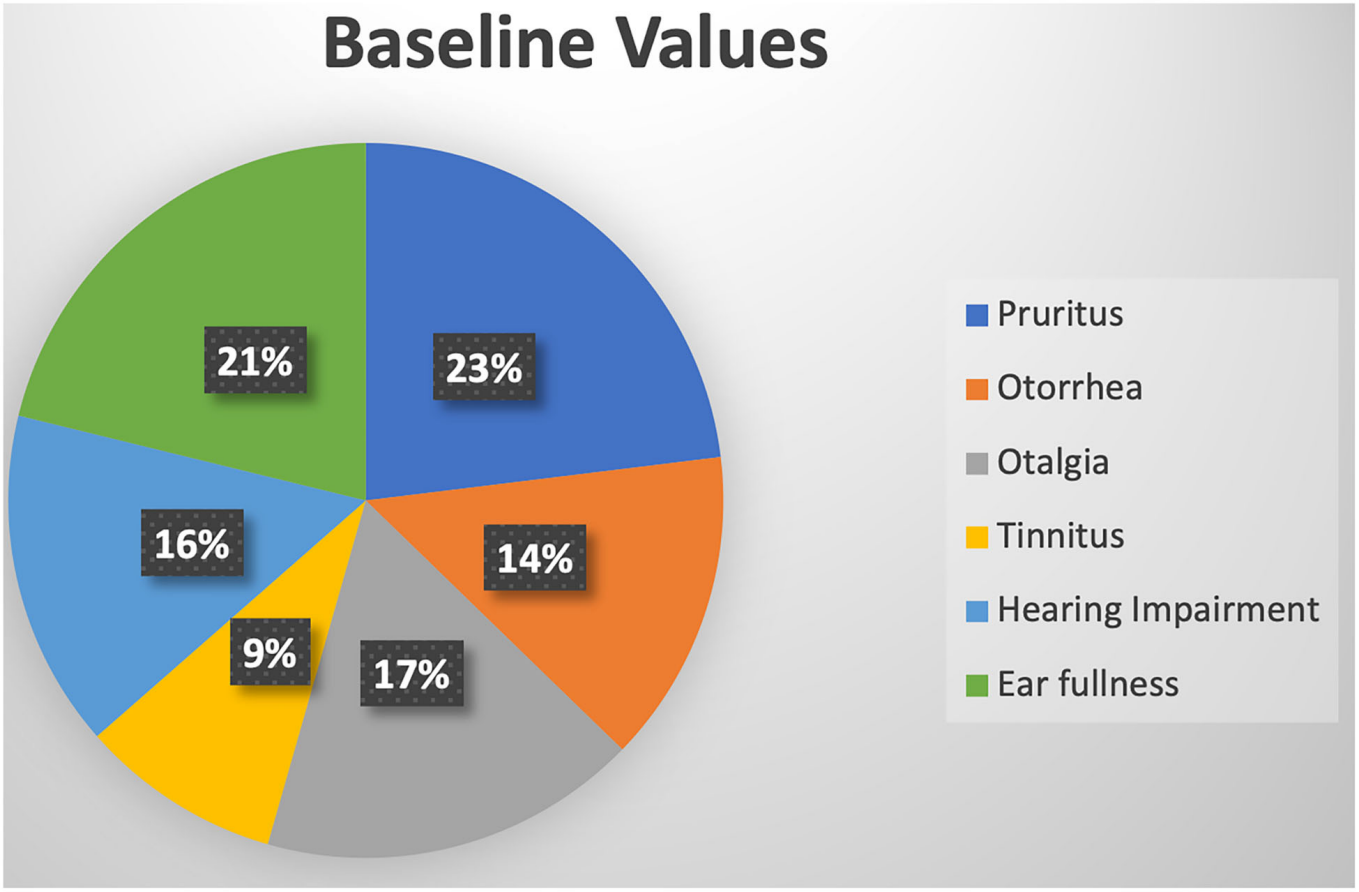
Ear symptoms before medication in the included studies.

The research areas included India, the Philippines, Nigeria, Iran, Thailand, and China. Most patients were located in hot and humid areas and economically underdeveloped areas. A total of 788 patients were included, including 375 males and 413 females. As shown in Figure 4, there was a female predominance, but there was a certain error due to the small sample size. After baseline ear symptom data were converted using the standard ratio, it was observed that the symptoms of ear itching and ear swelling in patients with external auditory canal mycosis were most common, and the symptom of tinnitus was relatively rare (Figure 5).

### Efficacy and Complications

**Figure 6:**
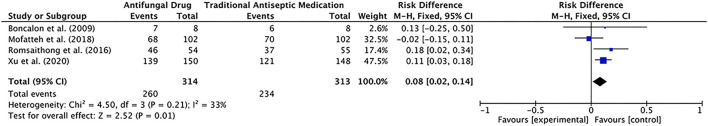
Forest plot of the efficacy of the two drug regimens analyzed in the meta-analysis.

**Figure 7:**
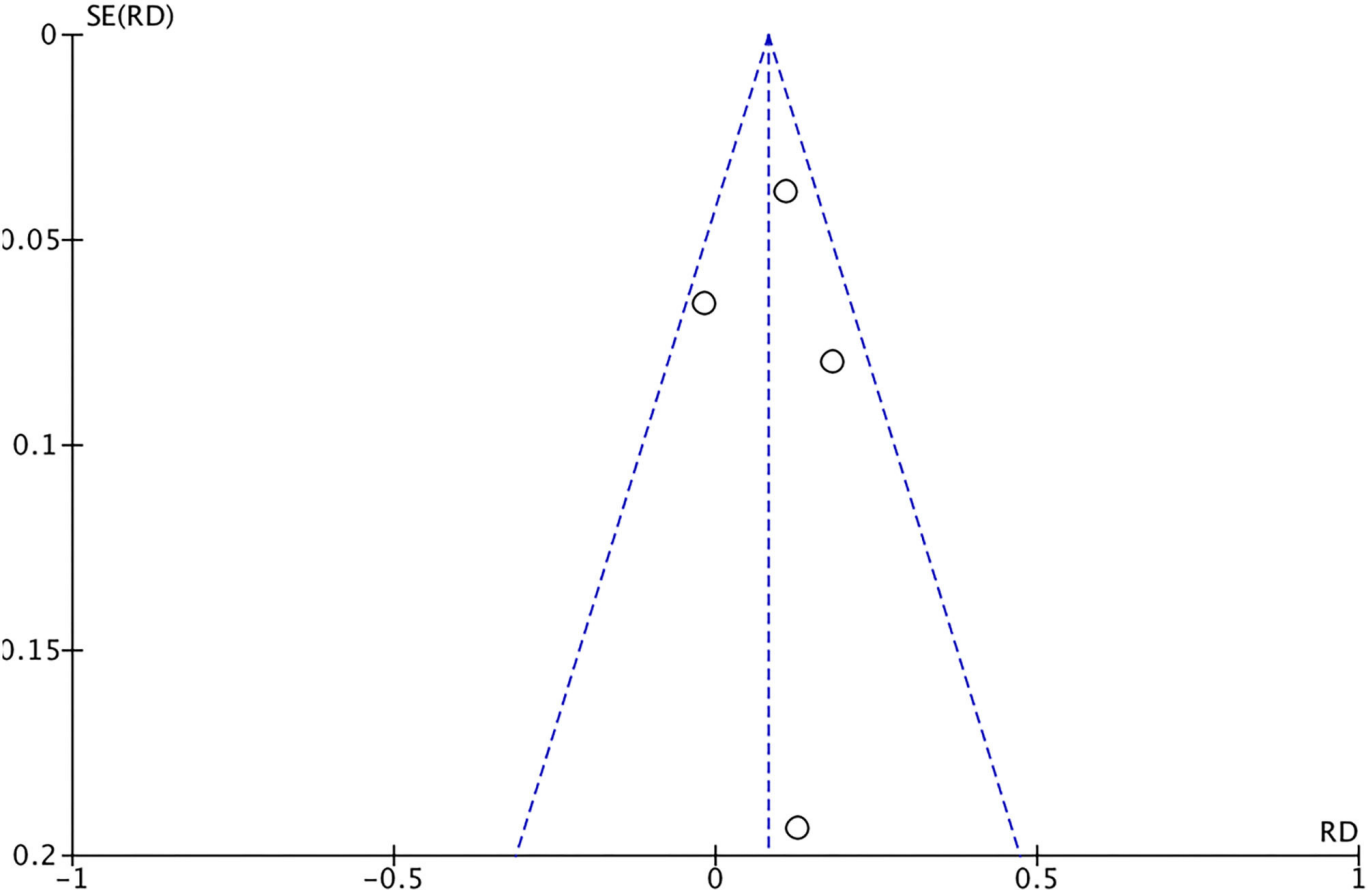
Funnel plot of the efficacy of the two drug regimens analyzed in the meta-analysis.

Four of the included studies described the relief of symptoms by two types of drugs. The antifungal group included 314 patients, of which 260 patients had complete or partial relief of symptoms after treatment. In contrast, the traditional antiseptic medication group included 313 patients, of which 234 patients had complete or partial relief of symptoms after treatment. The data were analyzed with a fixed-effects model (*P* = 0.21, I^2^ = 33%), and the results showed the following: total effect Z = 2.52, *P* = 0.01, RD = 0.08, 95% CI [0.02, 0.14]. The difference was statistically significant, suggesting that antifungal drugs were relatively effective (Figures 6, 7).

The comparison of six common complications after medication use by the fixed-effect model revealed the following: total effect Z = 1.76, *P* = 0.08, RD = −0.02, 95% CI [−0.04, 0.00]. The difference was not statistically significant. This result suggested that there was no significant difference in complications after the use of antifungal drugs or traditional antiseptic medication.

The authors further conducted subgroup analyses according to complications using fixed-effects models. The results indicated no significant difference in the rate of ear itching (Z = 0.02, *P* = 0.98), otorrhea (Z = 0.14, *P* = 0.89) or deafness (Z = 0.88, *P* = 0.38); that is, after medication use, both groups experienced complications of ear itching, otorrhea, and deafness, but the differences were not significant.

**Figure 8:**
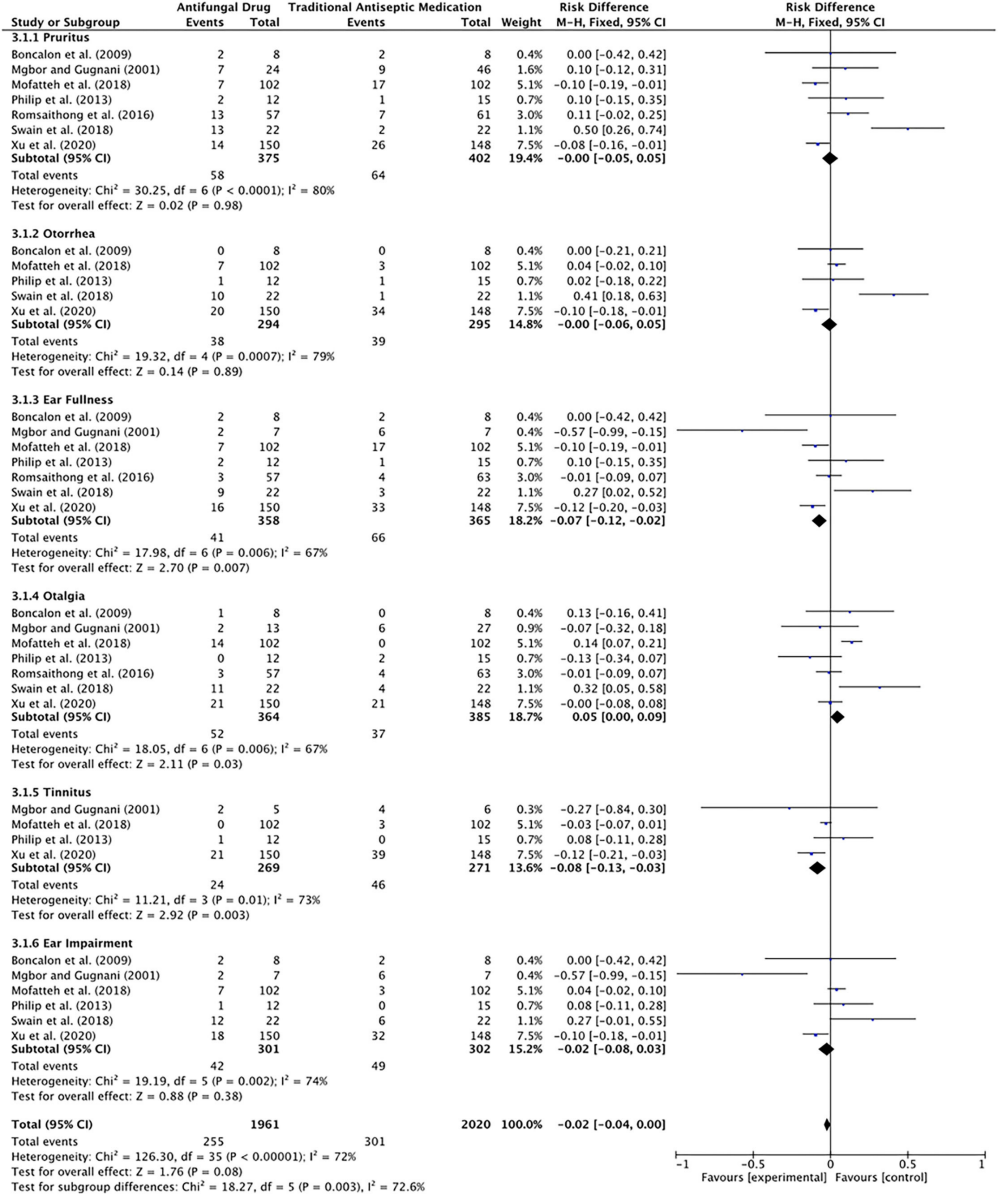
Forest plot of complications associated with the two drug regimens analyzed in the meta-analysis.

**Figure 9:**
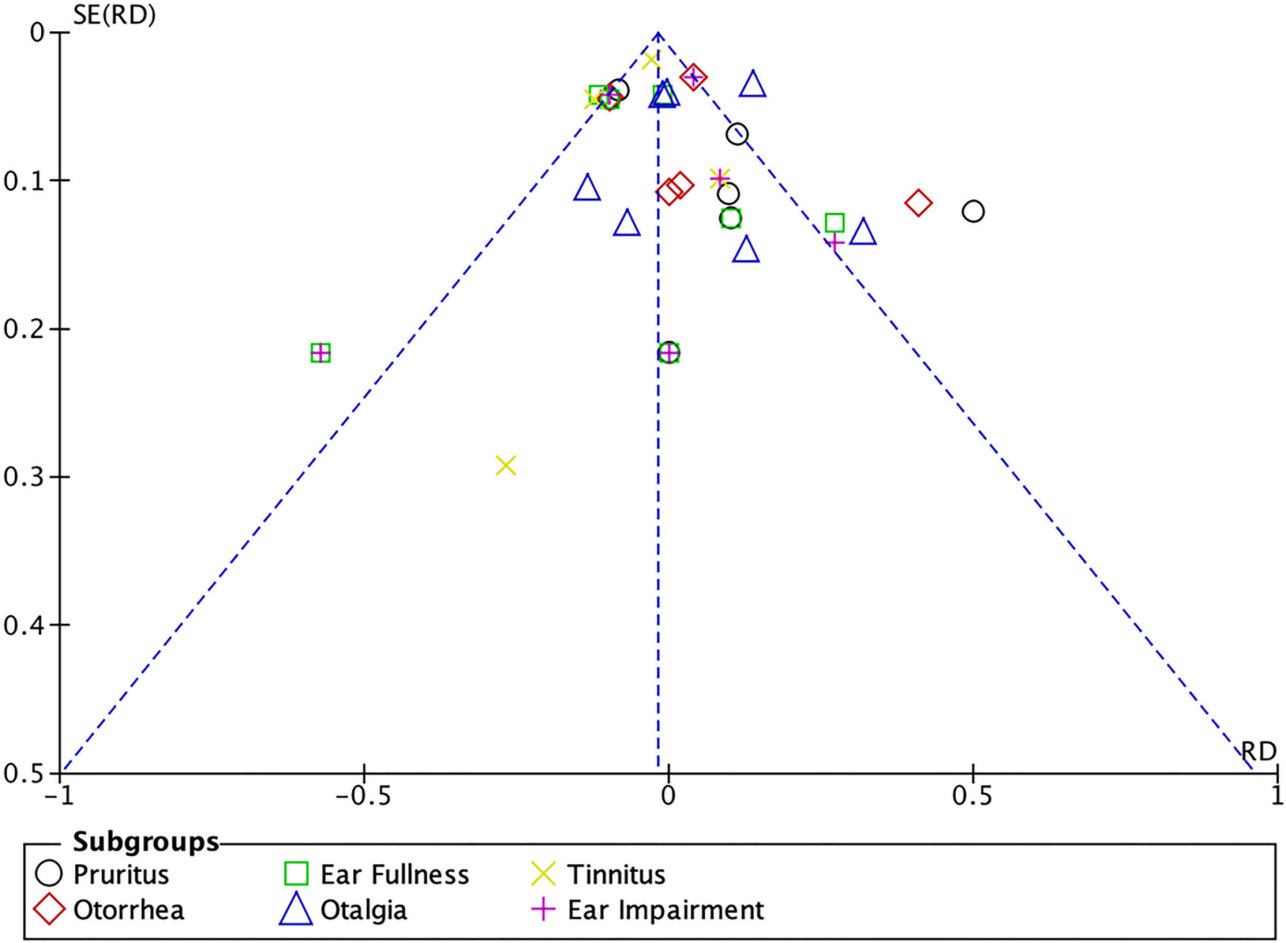
Funnel plot of complications associated with the two drug regimens analyzed in the meta-analysis.

The differences in the rates of ear distension (Z = 2.70, *P* = 0.007), earache (Z = 2.11, *P* = 0.03) and tinnitus (Z = 2.92, *P* = 0.003) were statistically significant. Patients who received antifungal drugs had lower rates of ear swelling and tinnitus, while patients who received non-antifungal drugs had lower rates of ear pain (Figures 8, 9).

## Discussion

Otomycosis is a superficial fungal infection that causes subacute or chronic inflammation due to fungal replication under certain conditions (18). The fungus invades the stratum corneum of the external auditory canal, causing otalgia, itching, hearing impairment, etc. This study analyzed current literature on the treatment of otomycosis with two types of commonly used clinical drugs. The results suggested that the therapeutic effects of the two types of drugs were statistically significant. Considering the easy application process and patient acceptance, the overall therapeutic effect of topical antifungal drugs was better than that of topical antiseptic medication; however, some treatments were incomplete. Traditional antiseptic medication, which is associated with complications such as tinnitus, nausea and vomiting after treatment, causes poor treatment adherence in some patients. The type of medication should be reasonably selected according to the condition of the disease.

This meta-analysis was conducted for three main reasons. First, the anatomical structure of the external auditory canal has a physiological curvature; therefore, different methods of medication application will have an impact on their efficacy (19). Antifungal drugs are generally cream preparations that partially cover the lesions, which are prone to recurrence. Non-antifungal drugs, by comparison, are generally liquid and penetrate deep into the external auditory canal, which not only reduces local inflammation and exudation but also completely covers the lesion (20). Second, antifungal drugs contain relatively tiny amounts of hormones, alleviating local inflammation and halting the proliferation of fungi more quickly than non-antifungal drugs. Therefore, antifungal drugs have better curative effects and lead to a faster recovery. Third, the included regions were mostly tropical and subtropical regions, indicating that the incidence of otomycosis has obvious regional characteristics. In addition, there were more female patients than male patients, which may be related to a weaker immune systems (21).

Limitations of this study should be noted. First, the included studies used various evaluation criteria to define cure, and some reported data that were unable to be used in this meta-analysis, leading to publication bias. Second, there was a lack of research conducted in North America and Europe. Finally, few relevant studies have been conducted in this field, and further analyses are needed.

Otomycosis, which is diagnosed by external auditory canal secretion smears, is a common and frequently occurring clinical disease (22). Once this disease is diagnosed, remedial measures should be implemented promptly. Two classes of drugs are currently available to treat this disease. The results of this study suggest the following. Antifungal drugs, such as clotrimazole, are associated with fewer complications and have a better cure rate but are also associated with poor treatment compliance and ear pain. Traditional antiseptic medications, such as boric acid alcohol, are associated with complications that seriously affect treatment compliance and completion. In summary, although this meta-analysis has certain shortcomings, it still has certain significance in guiding clinical drug treatment.

## Conclusion

This meta-analysis and literature review suggests that antifungal drugs and traditional antiseptic medication are effective in relieving symptoms in patients with otomycosis. These treatments also had a significant favorable effect on hearing. Overall, in the small number of studies evaluated, topical treatment was shown to be a safe, effective, and non-destructive alternative for patients who are refractory to initial medical therapy. Antifungal drugs had a better overall therapeutic effect with fewer complications; however, because of the obvious complications of earache, most patients found treatment unbearable. Traditional antibacterial medication is associated with a larger number of complications, although treatment is relatively thorough and inexpensive. Otolaryngologists have the option to use one medication or a combination of two drugs on the basis of the condition. However, these conclusions must be interpreted with caution due to the limitations of this analysis. Future research in this area should include RCTs with long-term follow-up to guide the development of guidelines for otomycosis to overcome some of the weaknesses noted in the literature.

## Data Availability Statement

The datasets presented in this study can be found in online repositories. The names of the repository/repositories and accession number(s) can be found in the article/supplementary material.

## Author Contributions

All authors listed have made a substantial, direct, and intellectual contribution to the work and approved it for publication. Shunyu Wu sorted data and wrote article. Yin Cheng provided information about literature search strategies, and assisted with the literature search. Shunzhang Lin assisted with the meta-analysis, and Huanhai Liu assisted with proofreading.

## Funding

This work was financially supported by a grant (No.0907) from the Department of Otolaryngological, Shanghai Changzheng Hospital, Shanghai, China and three grants from the National Science Foundation of China (Nos. 81541038, 81670905, and 81870702).

## Conflict of Interest

The authors declare that the research was conducted in the absence of any commercial or financial relationships that could be construed as a potential conflict of interest.

## Publisher's Note

All claims expressed in this article are solely those of the authors and do not necessarily represent those of their affiliated organizations, or those of the publisher, the editors and the reviewers. Any product that may be evaluated in this article, or claim that may be made by its manufacturer, is not guaranteed or endorsed by the publisher.

## Abbreviations

PRISMA: the Preferred Reporting Items for Systematic Reviews and Meta-Analyses
RCTs: Randomized controlled trials
NOS: the Newcastle Ottawa Scale
ORs: Odds ratios
Cis: confidence intervals.
